# Cleavage stage versus blastocyst stage transfers in patients with a single zygote: an emulated target trial

**DOI:** 10.1093/humrep/deag075

**Published:** 2026-05-29

**Authors:** Oisin Fitzgerald, Wentao Li, Catherine Vallence, Georgina M Chambers, Luk Rombauts

**Affiliations:** National Perinatal Epidemiology and Statistics Unit, Centre for Big Data Research in Health, University of New South Wales, Health Translation Hub, Randwick, Sydney, Australia; National Perinatal Epidemiology and Statistics Unit, Centre for Big Data Research in Health, University of New South Wales, Health Translation Hub, Randwick, Sydney, Australia; Fertility Consumer Advisory Group, University of New South Wales, Sydney, Australia; National Perinatal Epidemiology and Statistics Unit, Centre for Big Data Research in Health, University of New South Wales, Health Translation Hub, Randwick, Sydney, Australia; Monash IVF Group, Cremorne, VIC, Australia; Department of Obstetrics and Gynaecology, Monash University, Clayton, VIC, Australia

**Keywords:** assisted reproduction, statistics, embryo development, IVF/ICSI outcome, infertility

## Abstract

**STUDY QUESTION:**

Is the live birth rate higher for cleavage stage embryos compared to blastocysts in patients with a single zygote following oocyte fertilization using IVF or ICSI?

**SUMMARY ANSWER:**

Among patients with only a single zygote, transfer at cleavage stage was observed to result in a higher live birth rate than transfer at blastocyst stage.

**WHAT IS KNOWN ALREADY:**

Existing evidence suggests that blastocyst transfer is superior to cleavage stage in terms of live birth rate per embryo transfer, cumulative live birth rate, and time to pregnancy with three or more zygotes. However, whether these findings generalize to cohorts with less than three zygotes remains unclear.

**STUDY DESIGN, SIZE, DURATION:**

This target trial emulation, with live birth as the primary outcome, involved a retrospective analysis of 11 163 nulliparous patients who undertook ART in Australia and New Zealand between 2009 and 2022. Participants were included in the study if they were undergoing their first-ever stimulated ART cycle resulting in a single zygote following fertilization using IVF or ICSI. In this cohort, there were 6505 patients who received a cleavage stage transfer, 2216 who received a blastocyst stage transfer, and 2442 who had no embryo available for transfer following embryo culture, with the intended day of transfer unknown for these patients.

**PARTICIPANTS/MATERIALS, SETTING, METHODS:**

We modelled a comparison of intended transfer of a cleavage or blastocyst using g-computation within the target trial emulation framework. This involved fitting models that estimated the chance that embryo would survive to cleavage or blastocyst stage based on patient characteristics, and if it did so, the chance of a live birth when it is transferred. These models were used to simulate an idealized randomized controlled trial (target trial) on our retrospective cohort.

**MAIN RESULTS AND THE ROLE OF CHANCE:**

We found that compared to blastocyst transfers, cleavage stage transfers were associated with a higher live birth rate per couple in this cohort (12.5% vs 10.1%), with an adjusted relative risk of 1.24 (95% CI: 1.15–1.50). This effect increased with female age from 35 years, with a relative risk of 1.34 (95% CI: 1.15–1.57) in a 35-year-old and 1.51 (95% CI: 1.25–1.80) in a 40-year-old woman. This is likely due to the high rate of embryo attrition between the cleavage and blastocyst stages; our models predict that on average 92.0% of zygotes would survive to cleavage stage, compared to 58.9% and 49.9% surviving to the blastocyst stage at ages 35 and 40 years.

**LIMITATIONS, REASONS FOR CAUTION:**

The originally intended/planned day of embryo transfer, i.e. cleavage stage or blastocyst, is not recorded in the data source, only the actual day of transfer. This required utilization of a multinomial mixture model that estimates the cleavage and blastocyst embryo development rates as a sub-model, using the year of treatment as an external source of variation (in additional to patient factors) for predicting the intended treatment group. As with any causal analysis using retrospectively collected observational data, the results are dependent on the accuracy of our modelling assumptions which cannot be verified. Additionally, the potential confounder of embryo quality on the day of transfer was not available.

**WIDER IMPLICATIONS OF THE FINDINGS:**

These results highlight the role target trial emulation can play in filling evidence gaps for patient cohorts excluded from existing RCTs, and where the prospect for future RCTs is limited due to sample size constraints or ethical considerations. Further, the assessment of new ART technologies and procedures needs to be stratified by markers of patient prognosis (in this case, female age and number of available zygotes), and there should be caution in generalizing the findings to a different group.

**STUDY FUNDING/COMPETING INTEREST(S):**

This study was funded by the 2024 Ferring Australia Reproductive Medicine Research Grant Scheme. O.F., G.M.C., and L.R. are the listed investigators on this grant paid to UNSW Sydney. Ferring had no role in designing, analysing, interpreting, or reviewing the study. W.L. declares that they are a Human Reproduction Deputy Editor. L.R. declares consulting fees from Besins, Merck, and Organon, speaker’s fees from Besins, travel support from Gedeon Richter, and shares in Monash IVF Group (ASX: MVF). C.V. declares receipt of gift vouchers as honorarium for consumer input to the study as part of their membership of UNSW YourIVFSuccess/National Perinatal Epidemiology and the Statistics Unit Consumer Advisory Group. The remaining authors have no conflicts of interest to declare.

**TRIAL REGISTRATION NUMBER:**

N/A.

## Introduction

There have been a number of advances in ART since the first birth using ART in 1978 (Fasouliotis and Schenker, 1999). In the first few decades of ART, embryos were generally transferred at cleavage stage (Day 2/3) due to embryo culture media failing to support blastocyst (Day 5/6) development (Chronopoulou and Harper, 2015). Improvements in embryo culture media in the 1990s offered an opportunity for transfer to be delayed until blastocyst stage. Extended embryo culture enables non-invasive embryo selection through *in vitro* identification of embryos with slow development or degeneration, and allows closer alignment between the timing of the transfer and embryo implantation in natural conception (Gardner *et al.*, 1998). By the 2010s, blastocyst transfer had become the default in countries with advanced ART programs such as Australia and New Zealand where, as of 2020, more than 90% of transfers were at the blastocyst stage (Newman *et al.*, 2024).

The benefits of blastocyst over cleavage stage transfer are partly supported by randomized control trials (RCTs). These have shown that blastocyst transfers result in an 8–21% relative improvement in live birth rate per embryo transfer (Glujovsky *et al.*, 2022) and a 9.9% relative improvement in 1-year cumulative live birth rates (CLBRs) per fertilization procedure (Ma *et al.*, 2024). However, an evidence gap is that most RCTs have been restricted to good prognosis patients: those with five or more zygotes (Neuhausser *et al.*, 2020) or three or more cleavage stage embryos (Ma *et al.*, 2024). In patients with a low number of usable embryos, culturing to blastocyst can result in a scenario where there are no embryos available for transfer. This raises the question of whether some of these embryos may have survived *in vivo* if they were transferred at cleavage stage. Arguments for this position include the potential for mismatch between embryo culture and the environment it needs for cell replication, with embryo culture potentially increasing exposure to oxidative stress or reactive oxygen species (Neblett *et al.*, 2021).

This study compares outcomes of cleavage and blastocyst stage transfers in ART naïve patients with only a single zygote, using retrospective observational data from Australia and New Zealand, 2009–2022. To provide clarity around the causal and modelling assumptions underlying our analysis, we utilize the target trial emulation framework (Hernán and Robins, 2016).

## Materials and methods

### Data and cohort

The data for this study come from the Australia and New Zealand Assisted Reproduction Database (ANZARD), a clinical quality registry comprising information on all ART treatment cycles undertaken in Australian and New Zealand fertility clinics (Newman *et al.*, 2024). This study uses ANZARD data from treatments conducted in 2009–2022. This analysis investigates whether, in nulliparous patients with a single zygote undertaking their first ever ART cycle, the decision to transfer the embryo at the cleavage or blastocyst stage impacts their chance of a live birth.

Male–female couples who met the following characteristics were included in the study: (i) intending parenthood using their own sperm and oocytes; (ii) initiating their first-ever stimulated ART cycle between 1 January 2009 and 1 October 2022, with an administrative censoring date of 30 December 2022 (giving a minimum follow-up time of 90 days postovarian stimulation procedure for patients who freeze their embryo); (iii) having a single zygote available following IVF or ICSI; and (iv) being nulliparous.

Patients who met any the following characteristics were excluded from the study: (i) having total fertilization failure in their first stimulated ART cycle; (ii) using pre-implantation genetic testing (for all indications); (iii) using donor oocytes, donor sperm or a gestational carrier; (iv) undertaking ART with the purpose of embryo donation; (v) having frozen an embryo and not returning to thaw the embryo within 90 days; and (vi) having attended a clinic that exclusively (or near-exclusively - using a 1% cutoff rate) performed cleavage or blastocyst transfers (no treatment variation).

Our primary outcome was a live birth, defined as a birth meeting the World Health Organization (WHO) definition and of 20 weeks or more gestation or 400 g or more birthweight (Newman *et al.*, 2024). Clinical pregnancy was included as a secondary outcome. A clinical pregnancy was defined as fulfilling at least one of: (i) ongoing at 20 weeks; (ii) evidence by ultrasound of an intrauterine sac and/or foetal heart; (iii) examination of products of conception revealing chorionic villi; or (iv) a definite ectopic pregnancy diagnosed laparoscopically or by ultrasound (Newman *et al.*, 2024). Within ANZARD, a cleavage stage embryo is defined as an embryo aged 1–4 days old after fertilization, while a blastocyst is defined as an embryo aged 5–6 days old after fertilization.

From ANZARD, we extracted the following patient characteristics: female age, male age, infertility diagnosis (male infertility, tubal disease, endometriosis, other female infertility, and unexplained infertility). Treatment data included: number of oocytes collected at oocyte retrieval, site of sperm extraction, number of oocytes undergoing attempted fertilization, use of IVF or ICSI, fertilization rate, indicator for failed embryo development (i.e. no embryo transfer), fresh/frozen-thaw status of transferred embryo, stage of embryo transferred (cleavage or blastocyst), and the above listed study outcomes. Other variables included the year of treatment.

Descriptive statistics are presented as median [25th percentile; 75th percentile] for continuous variables and percentages for categorical variables, calculated for all patients, and based on the treatment received (cleavage stage embryo transfer, blastocyst stage embryo transfer, or no transfer).

### Target trial

To provide clarity around the design and generalizability of our study, we utilized the Target Trial framework in study design (Hernán and Robins, 2016), combining it with use of the Structural Causal Model and associated tools and notation (Pearl, 2009). Supplementary Table S1 outlines the target trial and connection with the emulation analysis. These frameworks aid in avoiding biases that results from naïve comparison of live birth rates per cleavage or blastocyst stage embryo transfer. Failure to account for the no transfer group would result in selection bias (Delgado-Rodriguez and Llorca, 2004). In the current study, this (survivorship) bias arises due to the conditioning on the survival of embryos to the blastocyte stage to be allocated to that arm. The time zero concept within the target trial emulation (Hernán and Robins, 2016) aims to address this bias by making explicit our emulated target trial’s randomization timepoint, in this case on Day 1 in patients with a single zygote (Supplementary Table S1), ensuring our analysis is linked to a clinically modifiable decision.

Gaps between the target trial and emulation analysis include: (i) a desire to estimate the intention to treat effect (Hollis and Campbell, 1999; Gupta, 2011), with treatment intention (whether a cleavage or blastocyst stage transfer was scheduled) not recorded in our dataset and thus selection/survivorship bias, and (ii) no data on the potential confounders (in order of likely importance): embryo quality, BMI, or socioeconomic factors. Our approach to addressing the first of these gaps is covered below.

### Embryo development and treatment assignment models to account for selection/survival bias

We used a multinomial mixture model with treatment year, a clinic random effect plus patient and treatment covariates being used to predict the probability a zygote will survive to a cleavage or blastocyst stage embryo suitable for transfer or cryopreservation. This mixture model had, as outcomes, the counts of patients with a usable cleavage stage embryo, a usable blastocyst stage embryo, and no usable embryo, with each outcome modelled as a mixture of sub-models for treatment assignment and the probability of a usable embryo at each stage. This model took advantage of the changing rates of patient assignment to cleavage and blastocyst transfer over time in Australia. Indeed, had treatment intention been observed then calendar time may have been usable in an instrumental variable analysis (Swanson, 2017). The covariates were: female age, male age, infertility diagnosis (male infertility, tubal disease, endometriosis, other female infertility, and unexplained infertility), number of oocytes collected at oocyte retrieval (categorized as [1–2; 3–4; 5; or more]), fertilization method (IVF or ICSI), fertilization rate (binned as [0–25%; 26–50%; 51–75%; 76–100%]), and use of testicular sperm; the final forms of these models was determined using forward/backward variable selection using the Akaike Information Criterion (AIC) (Akaike, 1974) as the selection metric. In each submodel, ART clinic was modelled as a random intercept. Further details on the multinomial mixture model are provided in Supplementary Data File S1.

### Embryo transfer outcome models

The embryo transfer outcome models predict the probability of study outcomes (live birth, clinical pregnancy) given when a Day 3 or Day 5 embryo transfer occurs. We modelled the probability of these binary outcomes given covariates using a generalized additive model (GAM) with L2 shrinkage on both the spline terms/continuous variables (female age and male age) and categorical variables (infertility diagnoses (male infertility, tubal disease, endometriosis, other female infertility, and unexplained infertility), number of oocytes collected at oocyte retrieval, fertilization method (IVF/ICSI), and use of testicular sperm). ART clinic was modelled as a random intercept. The appropriate degree of shrinkage was determined using generalized cross-validation (GCV), using the R package *mgcv* (Wood, 2000, 2017).

### Causal estimation

An overview of our causal modelling approach is provided in Supplementary Fig. S1. We utilized g-computation in the calculation of the treatment effects (Robins, 1986; Vansteelandt and Keiding, 2011). This involves using the embryo development and embryo transfer outcome models to simulate an idealized RCT on the cohort of patients meeting our target trial criteria. For each patient, we predict their expected potential outcomes under both scenarios of interest: assignment to receive a cleavage and assignment to receive a blastocyst transfer, with assignment following successful fertilization of one zygote (Day 1 of embryo growth). These patient level predictions are used to calculate the average treatment effect (ATE) both as a risk difference (RD) and risk ratio (RR) with blastocyst the reference group for each comparison. To investigate potential heterogeneity in the treatment effect, we additionally calculated the ATE conditional (CATE) on female age. This was done by fitting a GAM with the (difference or ratio of) patient level counterfactual predictions returned as part of the g-computation algorithm as the outcome and female age (as a spline term) as the inputs. Confidence intervals for the ATEs and CATEs were calculated using the bootstrap, with *B *= 1000 resamples of the data.

### Sensitivity analyses

We illustrate the sensitivity of our findings to changes in the underlying embryo survival and outcome models by graphing the results on a plot that varies the mean blastocyst development rate (embryos surviving from zygote to blastocyst) from 30% to 70% and the mean blastocyst live birth rate per embryo transfer from 5% to 25%, while keeping the cleavage development and live birth rate per embryo transfer fixed at the mean values from the study.

### Ethics

Approval for this project was obtained from the UNSW Sydney Human Research Ethics Committee (reference number: iRECS0859).

## Results

### Cohort

Table 1 provides an overview of the study cohort. Patients in the no transfer (median: 37.3 [IQR: 33.6; 40.6] years) and cleavage stage (37.0 [IQR: 33.1; 40.5] years) groups were ∼1 year older than the blastocyst stage group (36.3 [IQR: 32.5; 39.8] years). All groups had a similar number of oocytes retrieved and undergoing IVF/ICSI, with 50% of patients having between two and five oocytes retrieved and between one and four oocytes undergoing attempted fertilization. Fertilization rates per patient were non-symmetrical and skewed, with a mean fertilization rate per patient of 0.5 for all groups, although the blastocyst group had a median [IQR] fertilization rate per patient of 0.3 [0.2; 0.5]. There were more frozen-thaw transfers in the blastocyst stage group (8.3%) compared to the cleavage stage group (1.3%). Overall, just over 1 in 10 patients had a live birth (11.5%). In line with expectations, there was a higher per transfer clinical pregnancy and live birth rate in the blastocyst stage group (24.1% and 18.4%) compared to the cleavage stage group (18.4% and 13.5%).

**Table 1.** Overview of study cohort, comparison of cleavage and blastocyst stage transfer in patients with a single zygote.

Variable	All patients	Development stage of embryo		
		**No transfer**	**Cleavage stage**	**Blastocyst stage**
**Number**	11 163	2442	6505	2216
**Female age**	36.9 [33.1; 40.4]	37.3 [33.6; 40.6]	37.0 [33.1; 40.5]	36.3 [32.5; 39.8]
**Male age**	38.0 [33.0; 42.0]	38.0 [34.0; 42.0]	38.0 [34.0; 42.0]	37.0 [33.0; 41.0]
**Endometriosis**				
Yes	1423 (12.8%)	281 (11.5%)	879 (13.5%)	263 (11.9%)
No	9740 (87.3%)	2161 (88.5%)	5626 (86.5%)	1953 (88.2%)
**Tubal disease**				
Yes	828 (7.4%)	129 (5.3%)	543 (8.4%)	165 (7.0%)
No	10335 (92.6%)	2313 (94.7%)	5962 (91.7%)	2272 (93.0%)
**Other female infertility diagnosis**				
Yes	3628 (32.5%)	810 (33.2%)	2082 (32.0%)	736 (33.2%)
No	7535 (67.5%)	1632 (66.8%)	4423 (60.0%)	1480 (66.8%)
**Male infertility**				
Yes	3253 (29.1%)	588 (24.1%)	2094 (32.1%)	571 (25.8%)
No	7910 (70.9%)	1854 (75.9%)	4411 (67.8%)	1645 (74.2%)
**Unexplained infertility**				
Yes	2552 (22.9%)	588 (24.1%)	1356 (20.8%)	608 (27.4%)
No	8611 (77.1%)	1854 (75.9%)	5149 (79.2%)	1608 (72.6%)
**Number of oocytes retrieved**	3.0 [2.0; 5.0]	3.0 [2.0; 6.0]	3.0 [2.0; 5.0]	3.0 [2.0; 5.0]
**Number of oocytes undergoing IVF/ICSI**	2.0 [1.0; 4.0]	3.0 [1.0; 4.0]	2.0 [1.0; 4.0]	3.0 [2.0; 4.0]
**Sperm source**				
Ejaculate	10 679 (95.7%)	2331 (95.5%)	6202 (95.3%)	2032 (96.8%)
Testicular	484 (4.3%)	111 (4.5%)	303 (4.7%)	70 (3.2%)
**Fertilization method**				
IVF	5184 (46.4%)	1161 (47.5%)	2898 (44.5%)	1125 (50.8%)
ICSI	5979 (53.6%)	1281 (52.5%)	3607 (55.5%)	1091 (49.2%)
**Fertilization rate per patient**	0.5 [0.2; 1.0]	0.3 [0.2; 1.0]	0.5 [0.2; 1.0]	0.3 [0.2; 0.5]
**Transfer of a frozen-thaw embryo**				
Yes	290 (2.6%)	–	82 (1.3%)	184 (8.3%)
No	10873 (97.4%)	–	6423 (98.7%)	2032 (91.7%)
**Outcomes**				
**Clinical pregnancy rate**	15.5%	–	18.4%	24.1%
**Live birth rate**	11.5%	–	13.5%	18.4%

Data from Australia and New Zealand, 2009–2022.

### Embryo development and treatment assignment models

**Figure 1.**
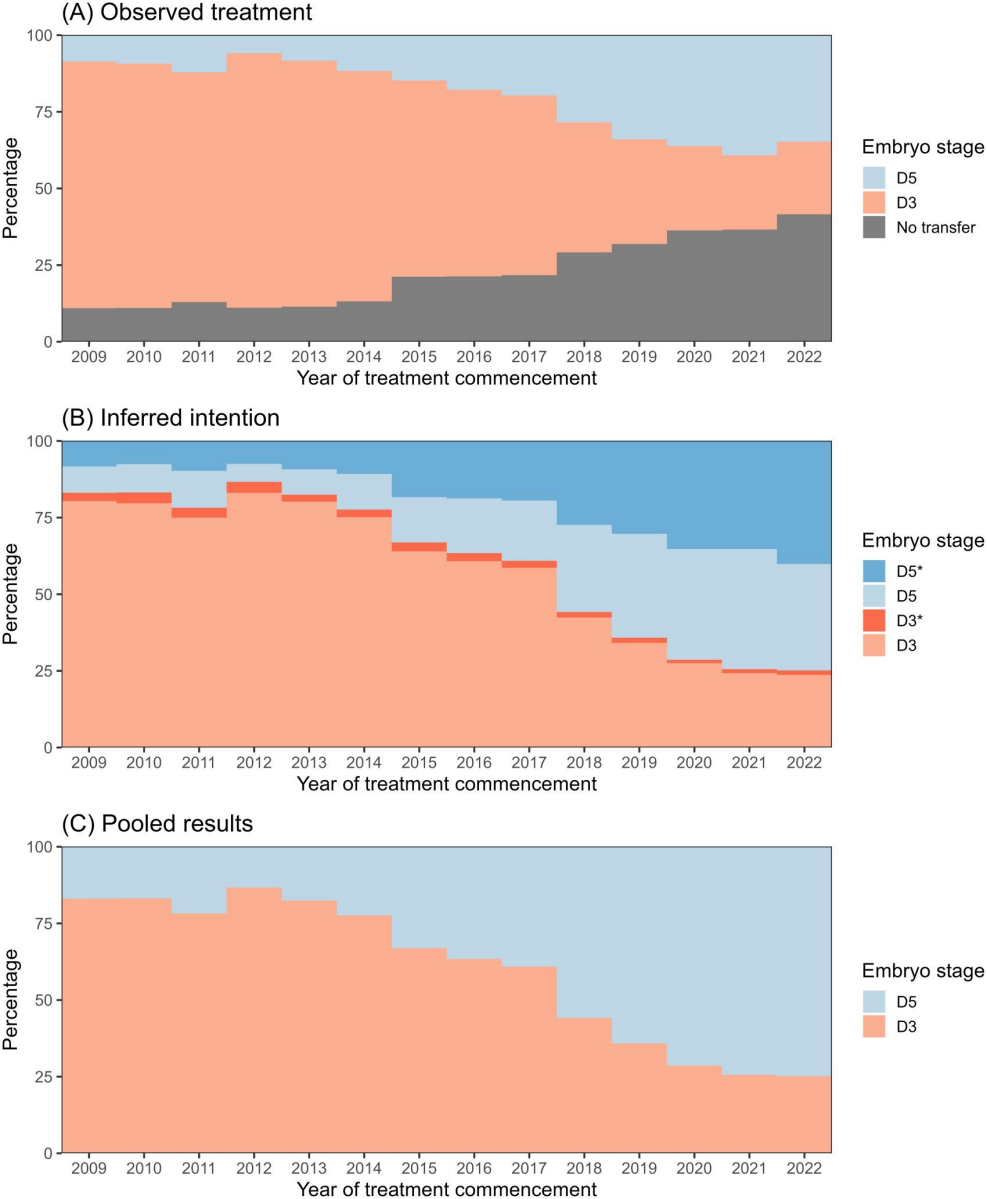
**Stage of embryo development at transfer in patients with a single zygote, Australia and New Zealand 2009–2022.** (**A**) Observed stage of embryo at transfer including no transfer group. (**B**) Observed and inferred rates of intended cleavage and blastocyst transfer. D3: cleavage stage; D3*: inferred intended cleavage stage transfer; D5: blastocyst stage; D5*: inferred intended blastocyst stage transfer. (**C**) Pooled results.

The final form of the embryo development models, following variable selection using AIC are shown in Supplementary Table S2. Following forward/backward variable selection using the AIC criterion, diagnoses of male infertility, tubal disease, and unexplained infertility and fertilization method (IVF/ICSI) were selected for inclusion in the model for cleavage stage development with ART clinic as random effect. The average failure rate for a zygote to develop to a cleavage stage was embryo estimated as 8.0%. These results are partially in line with a large study of 42 074 embryos where female age and fertilization method (IVF/ICSI) did not predict cleavage rate (∼90%) (Grøndahl *et al.*, 2017). The odds of failure to develop to a cleavage stage embryo were reduced in those with male infertility (aOR: 0.52 [0.40–0.69]), tubal disease (aOR: 0.32 [0.15–0.65]), unexplained infertility (aOR: 0.59 [0.42–0.81]) and increased with use of ICSI compared to IVF (aOR: 1.24 [0.98–1.57]). The following variables were included in the model for blastocyst stage development: female age, sperm source (testicular/ejaculate), and number of oocytes retrieved, with ART clinic as random effect, with the odds of failure increasing with female age (about 10% every 2 years), use of testicular sperm compared to ejaculate (aOR: 1.32 [0.95–1.84]) and having more oocytes retrieved (see Supplementary Table S2, for the latter). The average failure rate for a zygote to develop to a blastocyst stage embryo was estimated as 44.3%. The following variables were included in the model for treatment group assignment to cleavage stage transfer: year of treatment (see Supplementary Fig. S2), fertilization rate (reduced chance with increased fertilization rate; see Supplementary Table S2), and presence of unexplained infertility (aOR: 1.22 [1.05; 1.42]). Figure 1 illustrates the impact of this process, demonstrating that most of the no transfer group are implicitly assigned to the blastocyst group due to the far higher embryo development failure rates between cleavage and blastocyst stage.

### Outcome models

The final forms of the outcome models, following variable selection using L2 (ridge) penalization are shown in Supplementary Tables S3 and S4 and Supplementary Figs S3 and S4. Variables whose coefficients were shrank to zero (<0.01 on log-odds scale) (or equivalent for continuous variables) are not listed in the tables. Broadly, the results were as expected, with female age most strongly associated with the chance of a live birth and clinical pregnancy (Supplementary Figs S3A and S4A). The effects of all infertility diagnoses were weak, and even when the coefficient was not shrunk to zero (coefficient on log-odds scale <0.01), all confidence intervals around infertility diagnosis coefficients included the null (no effect). Retrieval of more than five oocytes was associated with a reduced chance of a live birth (aOR: 0.82 [0.71–0.96]) and clinical pregnancy (aOR: 0.83 [0.73–0.95]) compared to retrieval of 1–2 oocytes. We converted the number of oocytes retrieved from a continuous spline term to a categorical variable after examining a plot of the spline term. Transfer of a cleavage stage embryo was associated with a reduced chance of a clinical pregnancy (aOR: 0.74 [0.66–0.84]) and live birth (aOR: 0.74 [0.64–0.85]). Assessment of whether a treatment group differed by female age interaction effect was not warranted (reduction in deviance explained: 0.02%).

### Estimated treatment effects

Table 2 details the estimated treatment effects with planned cleavage stage transfer being superior for both outcomes. In particular, cleavage stage transfers were associated with a higher live birth rate per couple in this cohort (12.5% vs 10.1%), with an adjusted relative risk of 1.24 (95% CI: 1.15–1.50).

**Table 2.** Estimated effect of planned cleavage compared blastocyst stage (control) transfer on live birth and clinical pregnancy per patient in patients with a single fertilized oocyte undertaking their first ever ART treatment cycle.

Outcome	Cleavage stage	Blastocyst stage	Risk difference (95% CI)	Ratio (95% CI)
**Live birth rate per patient**	12.5%	10.1%	2.4% (1.6–4.4%)	1.24 (1.15–1.50)
**Clinical pregnancy rate per patient**	17.0%	13.4%	3.6% (2.7–6.2%)	1.27 (1.20–1.53)

Data from Australia and New Zealand, 2009–2022. CI: confidence interval.

### Impact of female age

**Figure 2.**
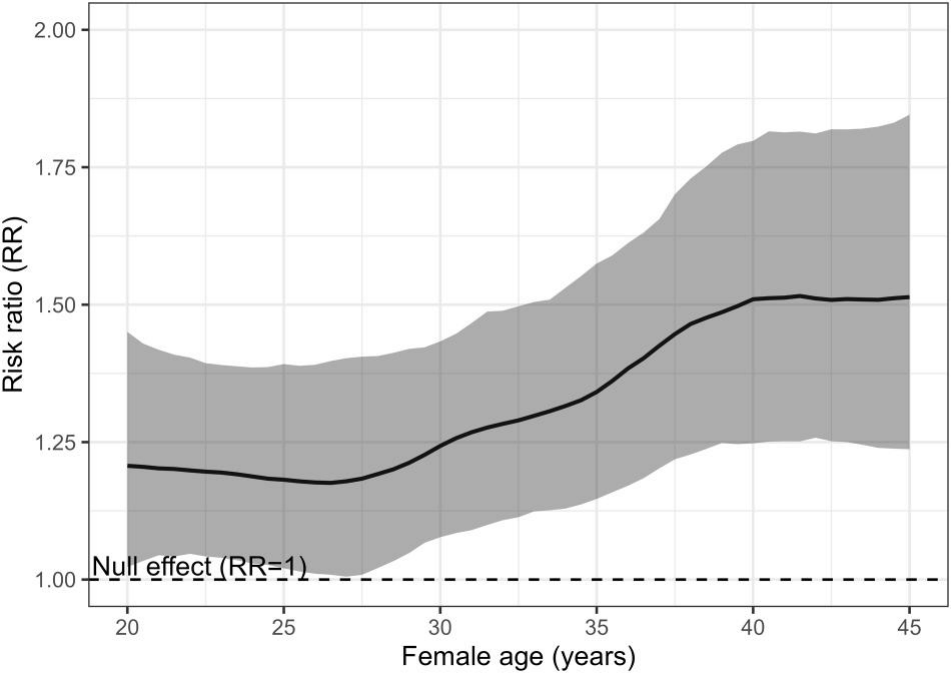
**Estimated effect (risk ratio with 95% confidence interval) of planned cleavage compared to blastocyst stage (control) transfer on live birth by female age at ovarian stimulation.** Patients were those with a single fertilized oocyte undertaking their first ever ART treatment cycle. Data from Australia and New Zealand, 2009–2022.

As shown in Fig. 2, the average treatment effect (on the risk ratio scale) was larger in women over 35 years. A 35-year-old with a single zygote had a relative increase in their chance of live birth with planned cleavage rather than blastocyst transfer of 1.34 (95% CI: 1.15–1.57) while the relative risk was 1.51 (95% CI: 1.25–1.80) in a 40-year-old. A large driver of this was embryo development: our embryo development models predicted that 92.0% of zygotes would survive to cleavage across all ages, while only 58.9% and 49.9% of zygotes survived to blastocysts among those aged 35 and 40 years.

### Sensitivity analysis

**Figure 3.**
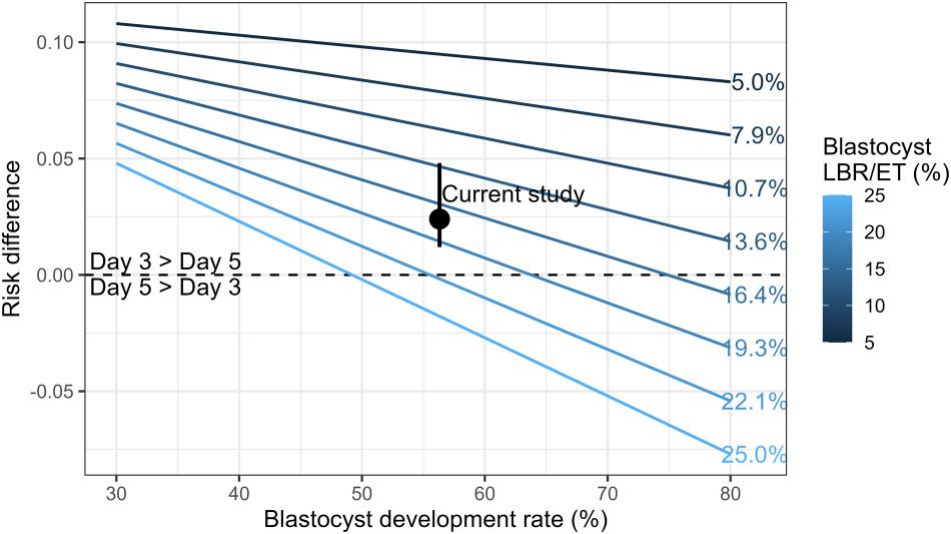
**Impact of varying mean blastocyst development rate and live birth rates per embryo on the calculated treatment effect.** Cleavage stage development rate is fixed at the estimate of 95.6% and live birth rate per cleavage stage embryo transfer is fixed at the population prediction of 13.0%. The blastocyst live birth rate per blastocyst transfer is the predicted live birth rate had all the patients received a blastocyst transfer. Patients were those with a single fertilized oocyte undertaking their first ever ART treatment cycle, Australia and New Zealand, 2009–2022. LBR/ET: live birth rate per embryo transfer.

The results appeared relatively robust to variations in mean blastocyst development (utilization) rate and live birth rate per embryo transfer. As shown in Fig. 3, the following would be required to nullify the study findings: (i) a 5% (absolute) increase in the blastocyst live birth rate per embryo transfer from 17% to 22%, or (ii) a 20% (absolute) increase in the blastocyst development rate from 56% to 74%. (Note that this graph is calculated by assuming that development rates and live birth rates per embryo transfer are constant across patients. This approximation introduces a small amount of error, around ∼0.004 in the live birth rate figures (Casella and Berger, 2024), which is too small to impact interpretation.)

## Discussion

This study compared outcomes of cleavage and blastocyst stage transfers in ART naïve patients with a single zygote using the emulated target trial methodology (Hernán and Robins, 2016), and specifically g-computation (Pearl, 2009; Hernán and Robins, 2020). This involved fitting models that estimated the chance an embryo would survive to cleavage or blastocyst stage based on patient characteristics, and if it did so, the chance of a live birth when transferred. These models were used to simulate an idealized experiment on the cohort of patients outlined in Table 1. We found that cleavage stage transfers were associated with a 24% relative higher live birth rate per patient/couple in this cohort (12.5% vs 10.1%). This effect was magnified in women over 35 years (Fig. 2) due to the reduced chances of an embryo surviving to the blastocyst stage, with the relative risk of live birth following cleavage stage transfer compared to blastocyst transfer being 1.34 (95% CI: 1.15–1.57) in a 35-year-old and 1.51 (95% CI: 1.25–1.80) in a 40-year-old.

The methods we utilized to perform the target trial emulation (synthetic experiment) (Pearl, 2009; Hernán and Robins, 2016, 2020) are based on the straightforward mathematical relationship between the live birth rate per embryo transfer and likelihood an embryo will survive to transfer. The per couple live birth rate is the product of these terms. If, as in the current sample, approximately half of cleavage stage embryos survive to blastocyst then the (cumulative) live birth rate per blastocyst transfer compared to cleavage stage embryo transfer must be at least double per couple on average (assuming most embryos survive to cleavage stage even if they are poor prognosis). In Australia and New Zealand, for the general ART population, the fresh single embryo live birth rates per blastocyst transfer is 28.4% compared to 15.3% for cleavage stage, a relative difference of 1.86, suggesting that for many patients, this is approximately the case (Newman *et al.*, 2024).

In the current cohort, the relative differences in live birth rate per embryo transfer were much smaller than for the general ART population, at 18.4% for blastocysts and 13.5% for cleavage stage: a 1.37 relative difference. This suggests that within the current cohort embryos were likely often of poor prognosis and/or transferred at an earlier stage. The cleavage stage utilization rate in this cohort was 92.0%. This compares with expected development rates of ≥95% to the 2-cell stage and 45–70% to the 8-cell stage (ESHRE Special Interest Group of Embryology, 2017). Similarly, the blastocyst development rate of 54% is on the higher range of the expected blastocyst development rate of 40–≥60%, and higher than the reported good quality blastocyst rates of 42.3% (Zacà *et al.*, 2022). Prior research has suggested patients with a larger number of oocytes retrieved (and so zygotes) tend to have a higher chance of good quality embryos (Vermey *et al.*, 2019). While embryo quality was not available in the current study, the high development rates (higher than reported good quality rates) and the low live birth rates support the hypothesis that the single embryos in our study were of poorer quality than in the average ART cohort. A link between embryo number and prognosis highlights a need for further research before any move to exclusive blastocyst transfer for patients with fewer zygotes, and development of more personalized estimates of optimal stage of embryo transfer.

Observational studies comparing blastocyst over cleavage stage transfer in patients with few zygotes have found somewhat contradictory results. In a retrospective propensity score matched study of 1142 patients, De Croo *et al.* (2022) found no difference in the CLBR per oocyte collection cycle between fresh cleavage or blastocyst stage transfer in patients with four or fewer zygotes. In a study of 1384 patients with only a single cleavage stage embryo, Xiao *et al.* (2019) found far higher (9.7% vs 4.4%, *P *< 0.01) live birth rates in patients with a cleavage stage transfer compared to those grown on to blastocyst stage, reporting an adjusted odd ratio of 2.60. These findings add support to those of Xiao *et al.* (2019) that, in patients with a single zygote/cleavage stage embryo, culture to the blastocyst stage may result in a lower live birth rate.

The findings from this study can be used to inform evidence gaps where future RCTs are unlikely to be practical, with the understanding that causal inference in observational relies on unverifiable assumptions (Pearl, 2009). The treatment effect (risk difference) and underlying live birth rates per patient/couple suggest a sample size of more than 5000 patients would be required to detect the observed group differences (which may differ from an appropriately designed RCT target) at 80% power (two-sided α = 0.05; $p_{1}\;$= 0.125; $p_{2}$ = 0.101) (Chow *et al.*, 2017). RCTs are often criticized for their inability to estimate generalizable or individual-specific effects, as doing so requires even large sample sizes (and thus greater expense and logistical burden) (Deaton and Cartwright, 2018). Indeed, detecting heterogeneous treatment effects (such as that for female age, Fig. 2) can require sample sizes up to four times larger than main effects alone (Brookes *et al.*, 2004). For comparison, the most recent 2022 Cochrane review of studies comparing cleavage and blastocyst transfer contained 2219 patients for the live birth rate analysis (Glujovsky *et al.*, 2022). This underscores that real-world data, interpreted together with RCT data, is essential for advancing personalized medicine, with frameworks such as hierarchical evidence synthesis (Welton *et al.*, 2012) and effect transportability (Pearl and Bareinboim, 2011) indispensable.

The results of this research suggest that findings from RCTs such as the study by Ma *et al.* (2024) comparing cleavage and blastocyst stage transfers cannot necessarily be generalized to all clinical contexts. Patients and the specialists treating them need to have confidence that the best quality evidence applies to their situation. Because adequately powered RCTs in a poor prognosis populations will always remain very difficult to fund and recruit for, emulated trial designs may be the best alternative to provide real-world clinical guidance.

This study is not without limitations. We didn’t have data on embryo quality, which may have been able to improve the accuracy of the embryo development and per embryo transfer outcome models, and would also have provided important information around how the patients in our cohort compared to the general ART population. In practice, embryo quality information measured at cleavage stage could also be a driver of when transfer occurs: immediately for poor quality embryos or at blastocyst stage for good quality embryos. We do not believe lack of embryo quality data would alter the direction of our findings: an embryo scored as poor quality would more likely result in cleavage transfer rather than risk culturing to blastocyst, biasing downward our live birth rate per cleavage transfer. A related limitation is the higher rate of frozen-thaw transfers in the blastocyst transfer patients (8.3% vs 1.3%; Table 1); while any clear benefit of freeze-all embryo strategies on live birth rate per patient are debated (Zaat *et al.*, 2021; Wei *et al.*, 2025), our estimate of the live birth rate per blastocyst embryo transfer may be slightly biased upwards if such an effect exists in patients whose embryos survive the freeze-thaw process. A third limitation is that we have calculated an intention-to-treat effect (Gupta, 2011), but for patients with no embryo transfer (21.8%) have no recorded intention or stage of embryo development failure. We have used a multinomial mixture model to model the unobserved treatment assignment process to account for this. As shown in the simulation studies in Supplementary Data File S1, this method results in unbiased treatment estimates so long as our causal and modelling assumptions are correct and we have available a non-confounder variable or set of variables that encodes variation in the treatment assignment mechanism (in this case, calendar time). As with any causal analysis using observational data, the results are dependent on the accuracy of our modelling assumptions which cannot be verified (Pearl, 2009). Terms such as ‘target trial’ should be understood as communication devices or study design frameworks rather than indicating such analyses are as good as randomized.

These results suggest that any adoption of uniform blastocyst stage transfer in ART patients would be premature. In the current study of patients with a single zygote, cleavage stage transfers are broadly preferably when considering clinical pregnancy and live birth rate. More research is required, extending the cohort to couples with more than one zygote, and including factors such as perinatal outcomes. Indeed, more generally, more evidence is required on what factors are associated with treatment success in poor prognosis ART patients given they make up 9–43% of patients (depending on the definition; Drakopoulos *et al.*, 2020; Esteves *et al.*, 2021), and tend to be excluded from RCTs. The cycle of ‘hope and despair’ experienced by ART patients is exacerbated in poor prognosis patients who endure substantial emotional, physical, and financial strain, and make up a substantial proportion of patients.

## Supplementary Material

deag075_Supplementary_Data_File_S1

deag075_Supplementary_Figure_S1

deag075_Supplementary_Figure_S2

deag075_Supplementary_Figure_S3

deag075_Supplementary_Figure_S4

deag075_Supplementary_Table_S1

deag075_Supplementary_Table_S2

deag075_Supplementary_Table_S3

deag075_Supplementary_Table_S4

## Data Availability

Data are available on reasonable request to the study authors subject to maintenance of data security and patient privacy.
