# Supplementary material for: Cleavage stage versus blastocyst stage transfers in patients with a single zygote: an emulated target trial
Source: Hum Reprod. 2026 May 29;41(7):1106–14. doi: 10.1093/humrep/deag075 (PMC13334914; doi:10.1093/humrep/deag075)
Supplement: deag075_Supplementary_Data_File_S1 [file deag075_supplementary_data_file_s1.docx]

Supplementary Data File S1

Cleavage stage versus blastocyst stage transfers in patients with a single zygote: an emulated target trial

# Methods

## Observed and unobserved data

Our approach aims to model the probability a zygote (fertilised oocyte – day 1 embryo) will survival embryo culture (laboratory growth) to either a cleavage stage or blastocyst stage embryo suitable for transfer or cryopreservation. For each patient $i$ we observe

- $y_{0,i}$: the number of transferred or cryopreserved cleavage stage embryos,
- $y_{1,i}$: the number of transferred or cryopreserved blastocyst stage embryos, and
- $y_{\emptyset,i}$: the number of unused embryos.

The number of zygotes (fertilised oocytes – day 1 embryo) is

$$n_{i}=y_{0,i}+y_{1,i}+y_{\emptyset,i}$$

Each patient is assigned an unobserved treatment intention (stage of embryo transfer and cryopreservation) $Z_{i}\in\{0,1\}$ (0=cleavage stage,1=blastocyst stage), which may not equal the observed treatment received $A_{i}\in\{0,1,\emptyset\}$ (0=cleavage stage,1=blastocyst stage, ø=no unused embryo). We also observe covariates $X_{i}$ that influence treatment assignment and/or embryo development.

## Multinomial mixture model

We model the counts of usable (transferred or cryopreserved) embryos in three mutually exclusive categories: cleavage-stage, blastocyst-stage, or unused expressed as mixtures of a latent assignment mechanism (intended transfer at cleavage or blastocyst stage) and the likelihood of embryo survival to that stage. By parameterising the no usable embryos cases as a mixture of intended cleavage and blastocyst stage utilisations, we can model the data as observations from a Multinomial distribution:

$$\text{(}y_{0,i},y_{1,i},y_{\emptyset,i}|x_{i}\text{) \textasciitilde Multinomial}(n_{i}; \left[ \begin{matrix} p_{0,i}=\pi_{0,i}(1-\alpha_{i}) \\ p_{1,i}=(1-\pi_{0,i})(1-\beta_{i}) \\ {p_{\emptyset,i}=\pi}_{0,i}\alpha_{i}+(1-\pi_{0,i})(1-\beta_{i}) \end{matrix} \right] )$$

Where $\alpha_{i}=\alpha(x_{i})$ and $\beta_{i}=\beta(x_{i})$ are the covariate dependent failure rates for development of a zygote to blastocyst and cleavage stage, and $\pi_{0,i}=\pi_{0}(x_{i})$ is the stratum specific probability of assignment to $Z=0$ (cleavage stage transfer/cryopreservation). These are modelled as:

$$\alpha(x_{i})=\text{logit}^{-1}({\gamma_{\alpha}}^{t}\Phi_{\alpha}\left( x_{i} \right))$$

With an analogous expression for $\beta(.)$ and $\pi_{0}(.)$, with $\Phi$ the design matrix constructor for each outcome - we may have additional covariates predicting survival to blastocyst compared to cleavage stage and will have additional variables for $\pi_{0,i}$ to ensure identifiability (see below). These quantities are related to the treatment received variable $A$ as

$$\text{Pr}\left( A_{i}=1 | x_{i},z_{i} \right)=(1-z_{i})\left( 1-\alpha\left( x_{i} \right) \right)+z_{i}(1-\beta\left( x_{i} \right))$$

### Identifiability assumptions

A key assumption of the model is that there exists heterogeneity in treatment assignment $Z$ within stratum of individuals with similar covariate values. If $\pi_{0,i}$ is a constant across the population or co-varies with predictors of cleavage or blastocyst stage utilisation then the data will lack sufficient variability to disentangle whether the observations are the result of treatment assignment or embryo development failure. An example of a variable that can provide this variability is calendar time. This is illustrated in Figure 2A in the main manuscript, which shows that for patients with a single fertilised oocyte, the introduction of blastocyst stage transfer almost exactly doubled the number of patients with no embryos to transfer suggesting a development rate of ~50%.

### Estimation

We estimated the model using maximum likelihood using the R statistical software routine optim.

## Causal estimation

As we cannot directly estimate any models of the outcome given treatment intention we use the following decomposition:

$$E\left[ Y | do\left( Z=z \right) \right]$$

$$= \sum_{x} E[y|z,x]p(x)$$

$$=\sum_{x} \sum_{a} E[y|a,x]p(a|x,z)p(x)$$

This decomposition enables us to avoid needing estimates of $E[y|z,x]$, with $Z$ the partially missing treatment intention variable, replacing this quantity with estimates of the embryo survival rates for cleavage and blastocyst stage embryo (see below), and the expected outcome from transfer of a cleavage or blastocyst stage embryo should one be available. The assumptions underlying this decomposition are inferred from the assumed data generating process, illustrated in the directed acyclic graph (DAG) in Figure 1.


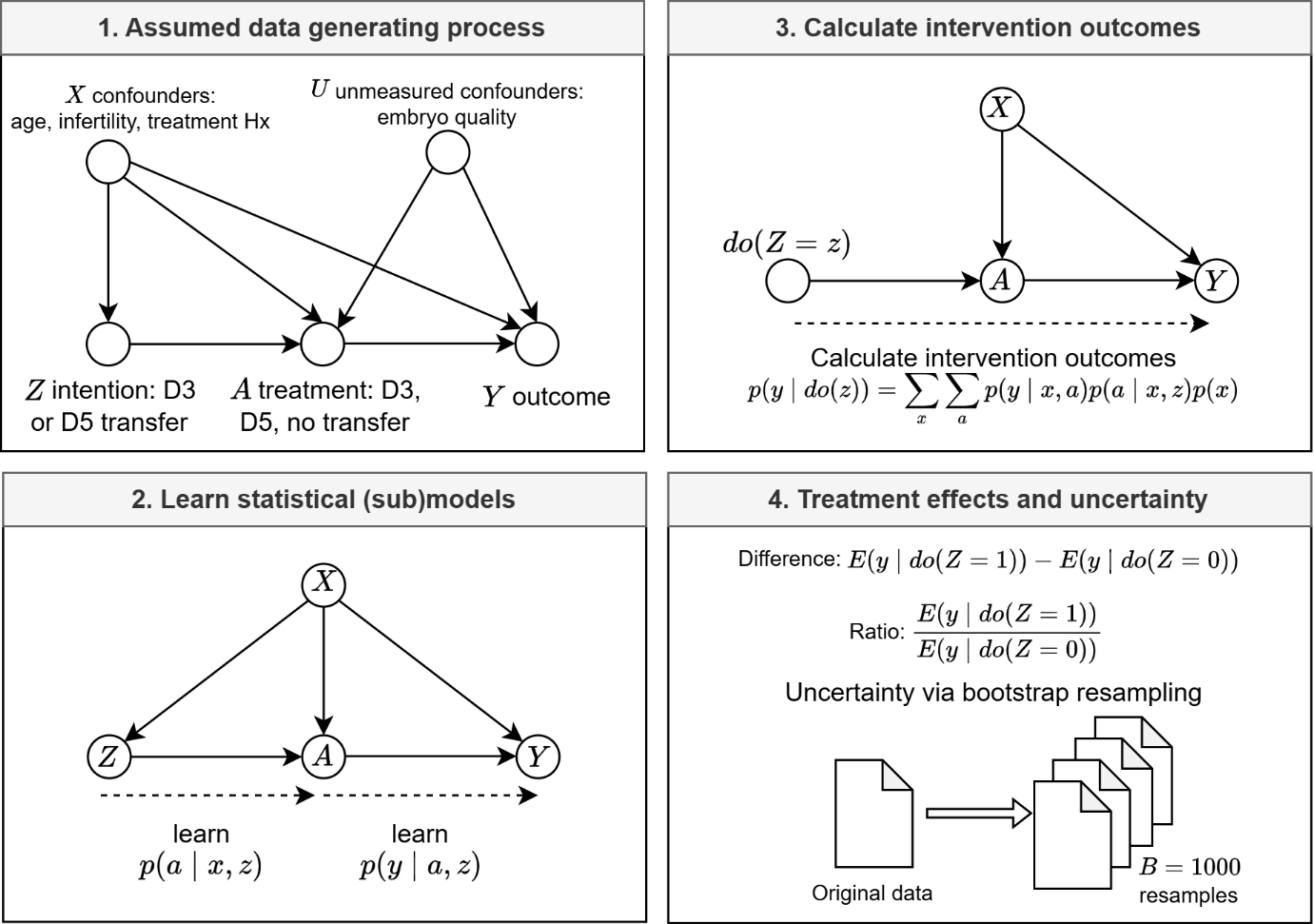


**Figure 1**. Overview of causal estimation procedure. (1) Assumed data generating process. (2) Estimation of sub-models connecting nodes in the graph. (3) Use of sub-models to perform synthetic experiments (4) Uncertainty calculated via the bootstrap.

## Simulation study

We assessed the bias of the methodology under three simulation settings, mimicking the real data in that the treatment intention was unobserved in each of the analyses and running each simulation $M=100$ times for sample sizes $n=1000, 2000, 5000, 10000$. Below, time *t* is a variable from 1 to 10 evenly distributed across the data.

### Setting 1

- Probability of assignment to intended cleavage stage transfer: $0.8-0.04t$
- 90% of embryos survive to cleavage stage and 50% to blastocyst
- Live birth rate per embryo transfer based on empirical data (female age and stage of embryo).

### Setting 2

- Probability of assignment to intended cleavage stage transfer: $0.75-0.04t+ 0.015(\text{female age} - 38)$
- 90% of embryos survive to cleavage stage and 50% to blastocyst
- Live birth rate per embryo transfer based on empirical data (female age and stage of embryo).

### Setting 3

- Assignment to intended stage of transfer random (50% chance of either group).
- Probability of survival to blastocyst: $0.5-0.005(\text{female age} - 38)$
- Probability of survival to cleavage stage: $0.88-0.003(\text{female age} - 38)$
- Live birth rate per embryo transfer based on empirical data (female age and stage of embryo).

# Results

As shown below the methodology does not appear to produce any systematically biased results, even in settings 2 and 3 where treatment assignment is non-random.

## Setting 1


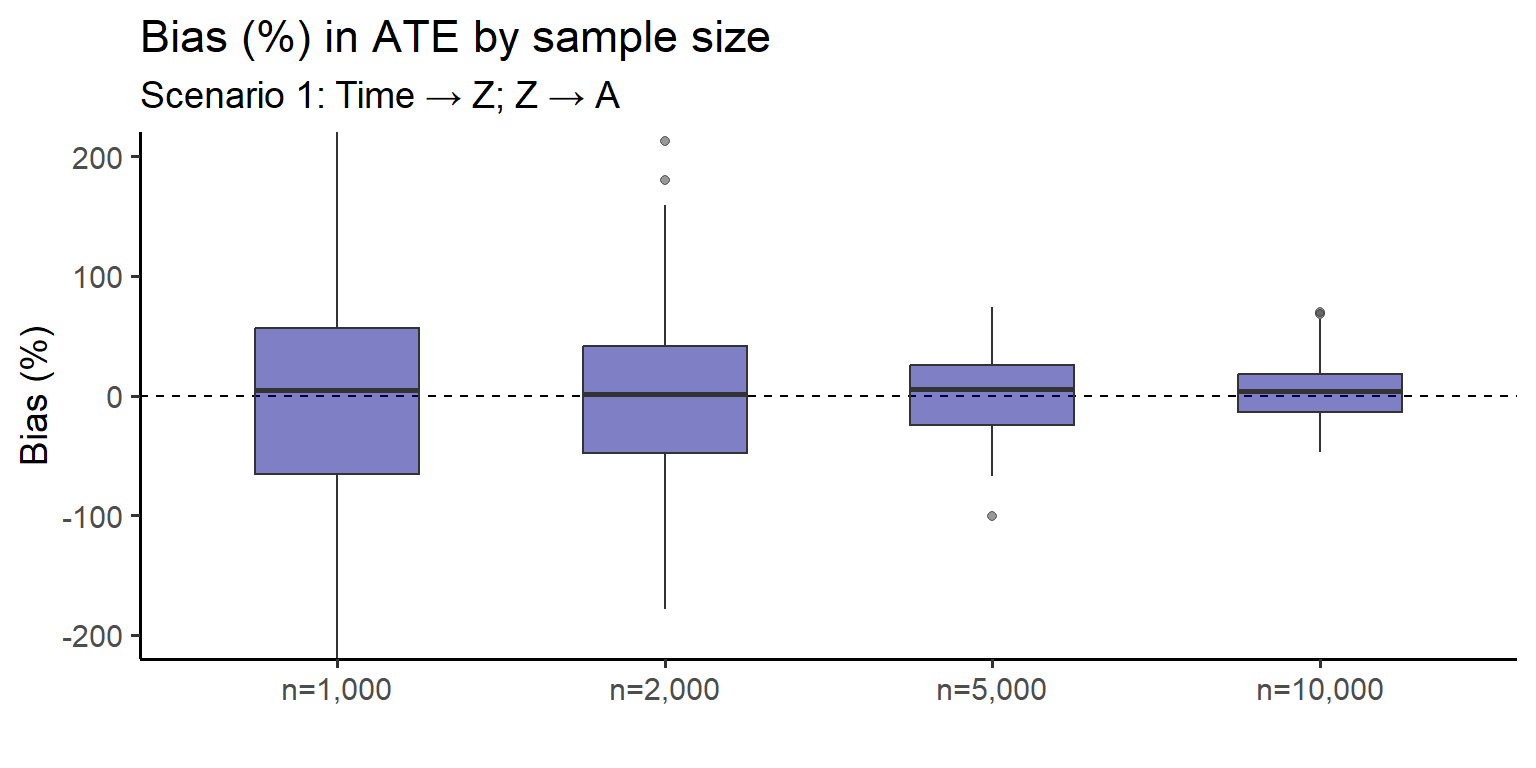


**Figure 2.** Percentage bias in the risk difference for *Setting 1* of a simulation study investigating a methodology for comparing intended cleavage and blastocyst stage transfer in data that does not record treatment intention. ATE: average treatment effect; Z: treatment assignment (unobserved); A: treatment received.

## Setting 2


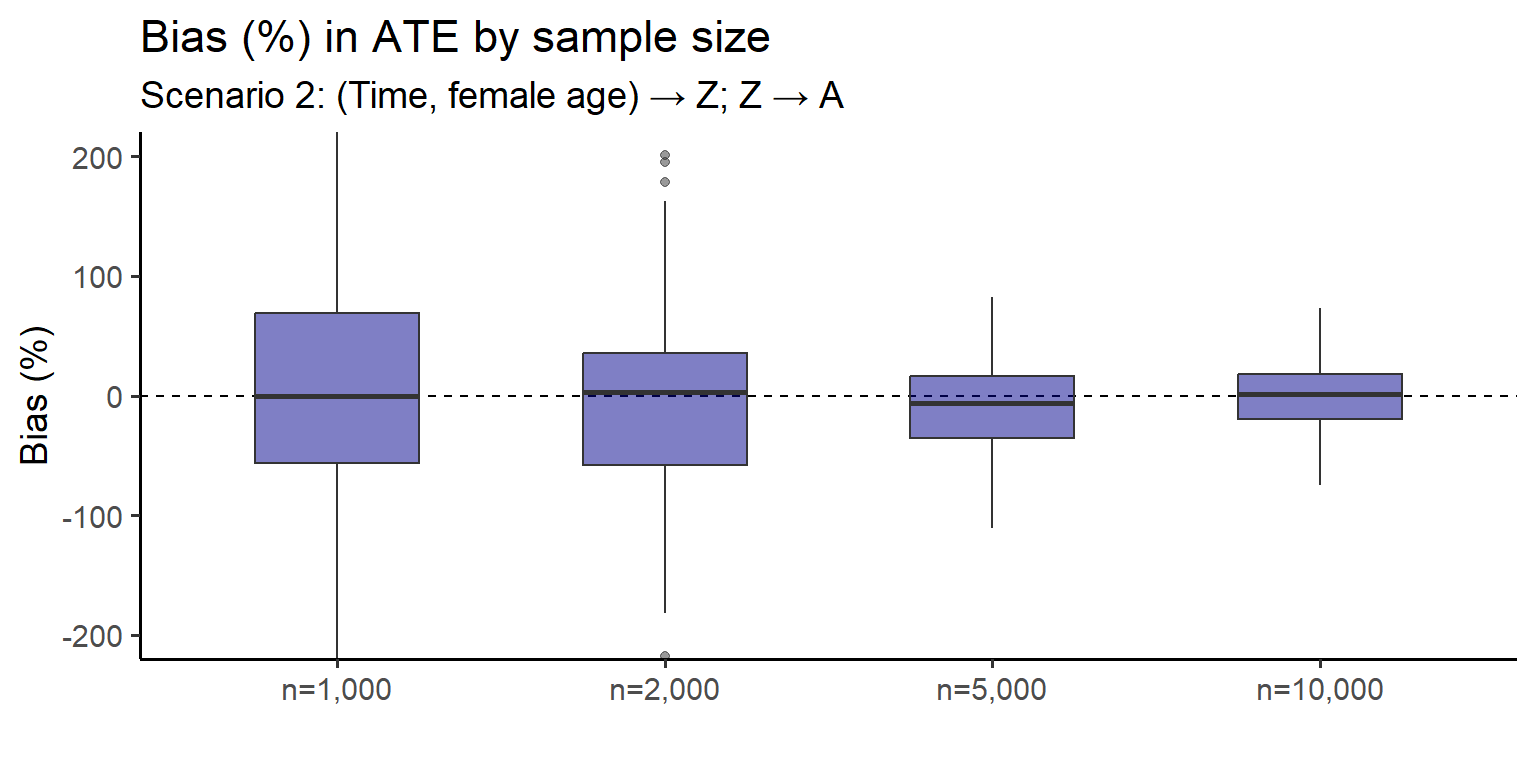


**Figure 3.** Percentage bias in the risk difference for *Setting 2* of a simulation study investigating a methodology for comparing intended cleavage and blastocyst stage transfer in data that does not record treatment intention. ATE: average treatment effect; Z: treatment assignment (unobserved); A: treatment received.

## Setting 3


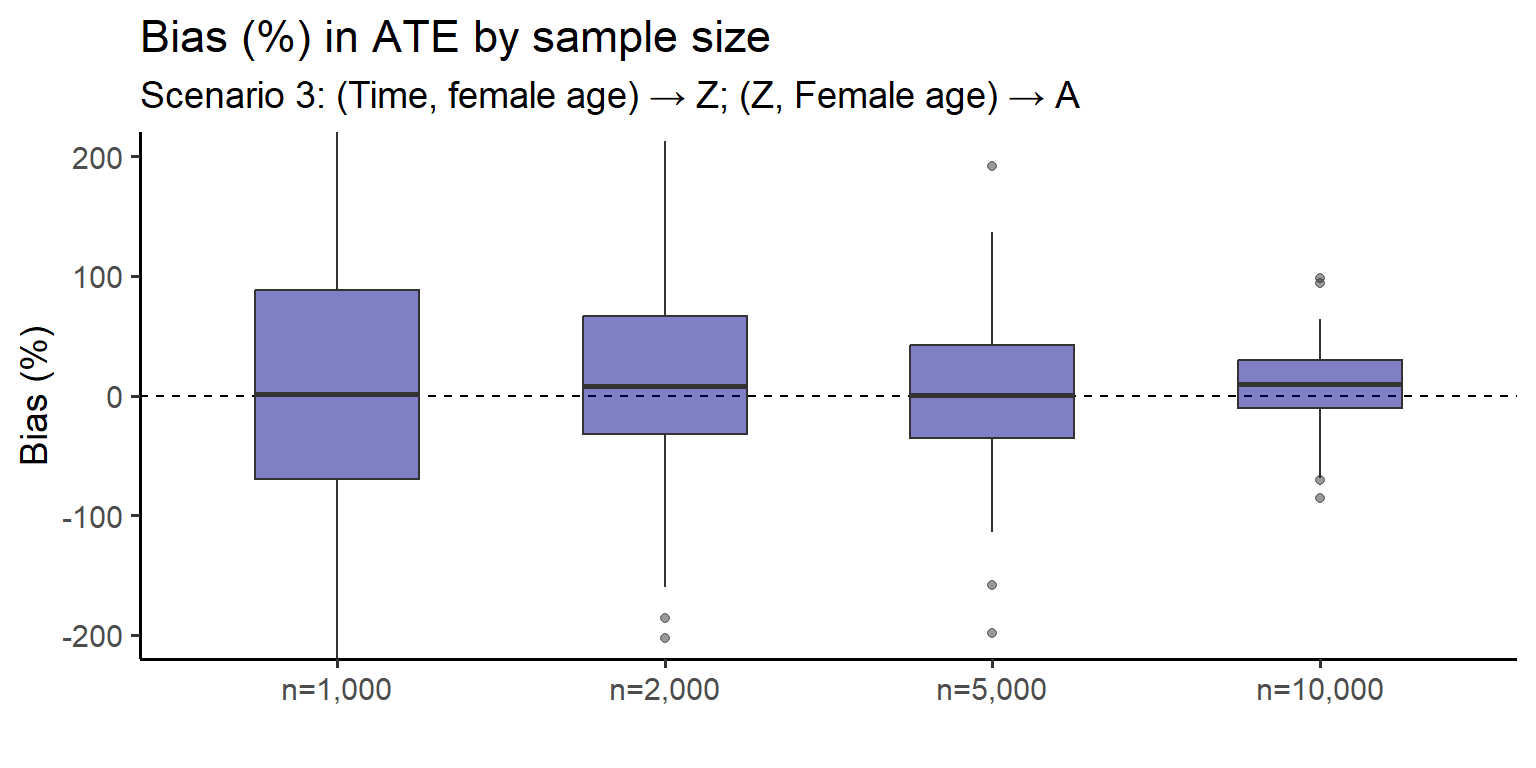


**Figure 4.** Percentage bias in the risk difference for *Setting 3* of a simulation study investigating a methodology for comparing intended cleavage and blastocyst stage transfer in data that does not record treatment intention. ATE: average treatment effect; Z: treatment assignment (unobserved); A: treatment received.
