# Supplementary material for: Cleavage stage versus blastocyst stage transfers in patients with a single zygote: an emulated target trial
Source: Hum Reprod. 2026 May 29;41(7):1106–14. doi: 10.1093/humrep/deag075 (PMC13334914; doi:10.1093/humrep/deag075)
Supplement: deag075_Supplementary_Figure_S1 [file deag075_supplementary_figure_s1.pdf]

Human Reproduction, 2026, 1–1
https://doi.org/10.1093/humrep/deag075
Supplementary Data

human reproduction

OXFORD

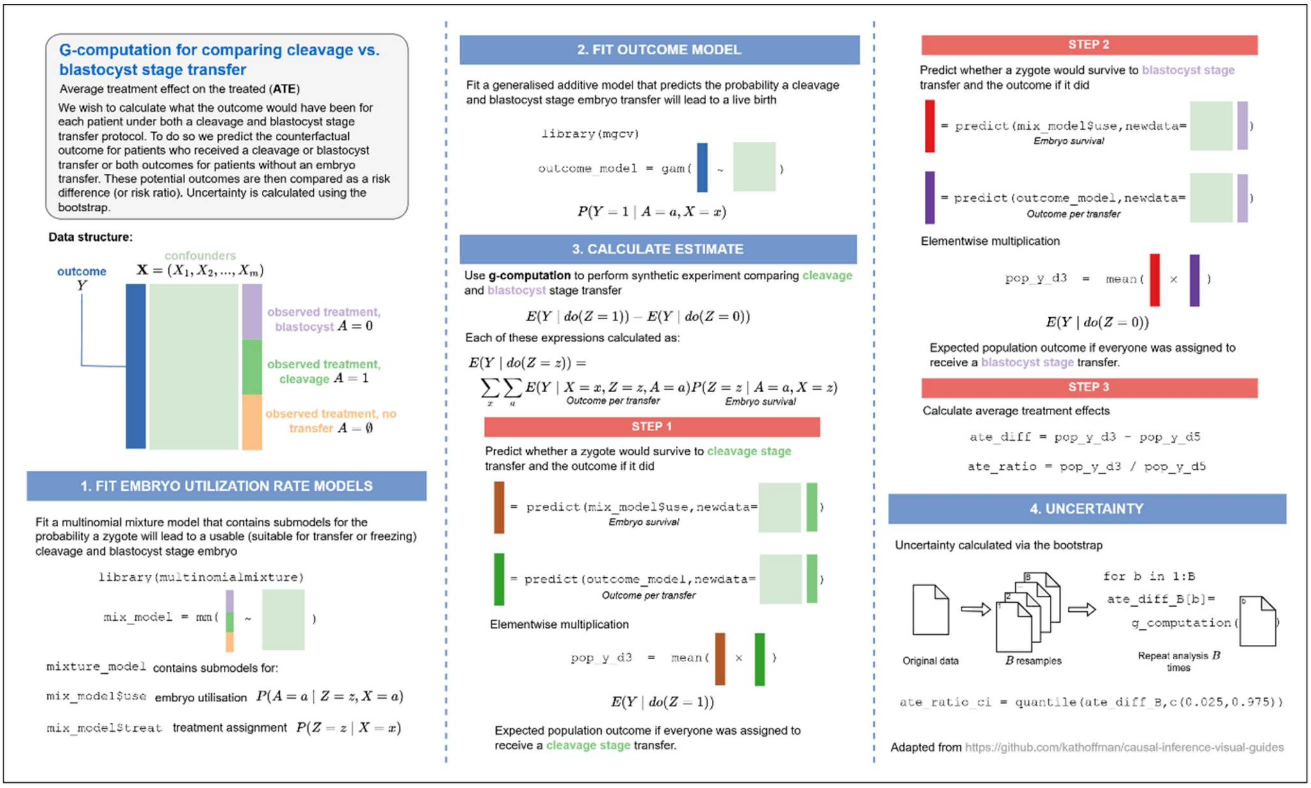

Supplementary Figure S1. Overview of statistical modelling and causal estimation steps in comparison of cleavage and blastocyst stage transfer in patients with a single zygote. Data were from Australia and New Zealand, 2009–2022.
