# Supplementary material for: Cleavage stage versus blastocyst stage transfers in patients with a single zygote: an emulated target trial
Source: Hum Reprod. 2026 May 29;41(7):1106–14. doi: 10.1093/humrep/deag075 (PMC13334914; doi:10.1093/humrep/deag075)
Supplement: deag075_Supplementary_Figure_S2 [file deag075_supplementary_figure_s2.pdf]

Human Reproduction, 2026, 1–1
https://doi.org/10.1093/humrep/deag075
Supplementary Data

human reproduction
OXFORD

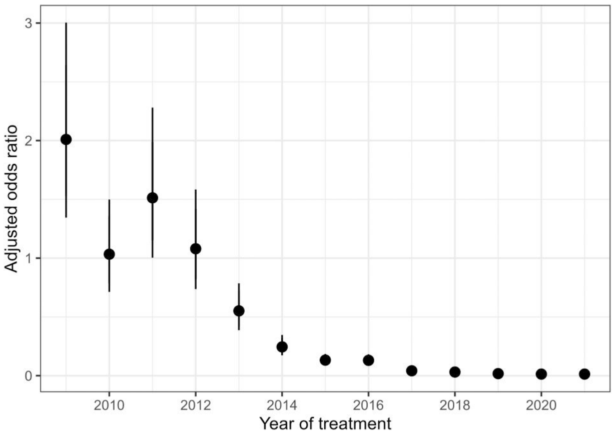

Supplementary Figure S2. Adjusted odds ratio of the effect of year of treatment on likelihood of being scheduled for a cleavage stage embryo transfer. Comparison of cleavage and blastocyst stage transfers in patients with a single fertilized oocyte, data from Australia and New Zealand, 2009–2022.
