# Supplementary material for: Cleavage stage versus blastocyst stage transfers in patients with a single zygote: an emulated target trial
Source: Hum Reprod. 2026 May 29;41(7):1106–14. doi: 10.1093/humrep/deag075 (PMC13334914; doi:10.1093/humrep/deag075)
Supplement: deag075_Supplementary_Table_S1 [file deag075_supplementary_table_s1.pdf]

Human Reproduction, 2026, 1–1
https://doi.org/10.1093/humrep/deag075
Supplementary Data

Supplementary Table S1. Target trial comparing cleavage and blastocyst stage transfer in patients with a single zygote.

Protocol component	Target trial	Emulation analysis
Eligibility criteria	Patients with one 2PN embryo (zygote) on day one following IVF or ICSI.<br>Patients must be nulliparous, and aged between 18 and 45 years old.<br>Patients are undertaking their first complete ART cycle (ovarian stimulation).<br>Treatments using donor oocytes or sperm or undertaken as part of a reciprocal IVF or surrogacy arrangement are excluded.<br>Treatments using pre-implantation genetic testing or assisted hatching are excluded.	Patients with one normally fertilized oocyte on Day 1 following IVF or ICSI. Normal fertilization is in the opinion of the treating embryologist. For example, in their opinion, although two pronuclei are not seen but cleavage has occurred, normal fertilization has occurred.<br>Other points as specification.
Treatment strategy	D3: Culture 2PN embryo to cleavage stage and transfer to patient<br>D5: Culture 2PN embryo to blastocyst stage and transfer to patient<br>Embryo freezing: All embryos should be transferred as part of a fresh ART cycle (i.e. within 6 days of fertilization) unless the following criteria are met in which case vitrification, thaw and transfer within 3 months may be considered:<br>· Risk of OHSS: e.g. based on E2 levels, PCOS AFC, or BMI<br>· Elevated Hormone Levels: E2/P ratio	**D3, D5:** ANZARD records the observed developmental stage (cleavage or blastocyst) of a transferred embryo, the occurrence of no transfer, or of embryo freezing and later thawing, but not the intention.<br>Embryo freezing: the data does not contain BMI, hormone levels, AFC, embryo quality, or stimulation protocol. We include embryo freezing and thaw cycles where the embryo is frozen, thawed, and transferred within 3 months assuming the decision to freeze was based on reasoning along the lines of our target trial.
Time 0	Day 1 of embryo growth, at the time when there was one zygote	As specification
Assignment procedure	Eligible patients will be randomly assigned to one of the treatment strategies and be aware of the strategy to which they were assigned.	We assume that patients who were recorded as receiving a cleavage and blastocyst stage transfer were in the **D3** and **D5** groups, i.e. that there was no intentional protocol deviation.<br>ANZARD does not explicitly record treatment intention, meaning this is completely missing information for the 20% of patients who have no embryo transfer.<br>Random assignment given:<br>· Female age<br>· Female BMI (not recorded)<br>· Cause of infertility<br>· Sperm extraction site<br>· Fertilization method IVF/ICSI)<br>· Embryo quality (not recorded)
Follow-up period	In the case of embryo freezing patients are followed for 3 months<br>All pregnancies will be followed to term.	As specification, ANZARD has $>$99% ascertainment of pregnancy outcomes.
Outcome	No transfer: percentage of cycles where no embryo transfer occurs<br>Clinical pregnancy: ANZARD definition<br>Live birth: ANZARD definitions	No transfer: not possible with ANZARD as it does not record intention, rather only whether a cleavage or blastocyst stage embryo was transferred.<br>Others as specification.

(continued)

human reproduction
OXFORD

Human Reproduction, 2026, 1–1
https://doi.org/10.1093/humrep/deag075
Supplementary Data
human reproduction

Supplementary Table S1. (continued)

Protocol component	Target trial	Emulation analysis
Causal contrast of interest	**Intention-to-treat effect:** effect of being assigned to a D3 or D5 treatment strategy<br>**Average treatment effect (ATE):** treatment effect across all patients (ITT)<br>**Conditional average treatment effect (CATE):** treatment effect within subgroups defined by female age.	As specification
Analysis plan	ATE calculated as average risk difference and risk ratio between groups, CATE estimated using generalized additive models	ATE calculated using g-computation. This involves fitting a multinomial mixture model that contains as sub-models for the chance an embryo would survive to cleavage or blastocyst stage based on patient characteristics, and models for the chance of a live birth (clinical pregnancy) given a cleavage or blastocyst transfer (and patient characteristics). These models are then used to simulate an idealized experiment on patients who meet the inclusion criteria.

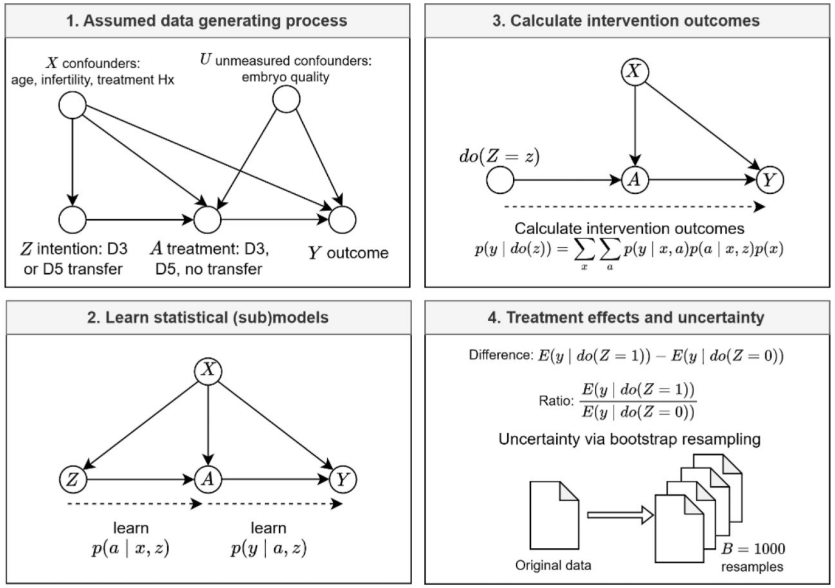

Data from Australia and New Zealand, 2009–2022.
