# Supplementary material for: Cleavage stage versus blastocyst stage transfers in patients with a single zygote: an emulated target trial
Source: Hum Reprod. 2026 May 29;41(7):1106–14. doi: 10.1093/humrep/deag075 (PMC13334914; doi:10.1093/humrep/deag075)
Supplement: deag075_Supplementary_Table_S2 [file deag075_supplementary_table_s2.pdf]

Human Reproduction, 2026, 1–1
https://doi.org/10.1093/humrep/deag075
Supplementary Data

Supplementary Table S2. Embryo development and treatment assignment models.

Variable	Adjusted odds ratio (95% confidence interval)
Failure to develop to Day 3 model	
Intercept	0.11 (0.09–0.32)
Male infertility	
No	0.0 (reference)
Yes	0.52 (0.40–0.69)
Tubal disease	
No	0.0 (reference)
Yes	0.32 (0.15–0.65)
Unexplained infertility	
No	0.0 (reference)
Yes	0.59 (0.42–0.81)
Fertilization method	
IVF	0.0 (reference)
ICSI	1.24 (0.98–1.57)
Failure to develop to Day 5 model	
Intercept	0.48 (0.38–0.61)
Female age	
18–30 years	0.0 (reference)
30–32 years	1.21 (0.92–1.59)
33–35 years	1.34 (1.03–1.73)
36–37 years	1.55 (1.19–2.02)
38–39 years	1.83 (1.39–2.40)
40–41 years	1.92 (1.46–2.53)
42–45 years	2.03 (1.53–2.68)
Source of sperm	
Ejaculate	0.0 (reference)
Testicular	1.32 (0.95–1.84)
Number of oocytes retrieved	
1–2	0.0 (reference)
3–4	1.07 (0.91–1.26)
5 or more	1.15 (0.99–1.35)
Planning a Day 3 transfer model	
Intercept	11.8 (8.70–15.87)
Year	See Supplementary Figure S1
Unexplained infertility	
No	0.0 (reference)
Yes	0.82 (0.70–0.95)
Fertilization rate	
0–25%	0.0 (reference)
26–50%	1.19 (1.00–1.41)
51–75%	1.34 (1.13–1.61)
76–100%	1.48 (1.24–1.76)

Comparison of cleavage and blastocyst stage transfer in patients with a single fertilized oocyte, data from Australia and New Zealand, 2009–2022.
