# Supplementary material for: Cleavage stage versus blastocyst stage transfers in patients with a single zygote: an emulated target trial
Source: Hum Reprod. 2026 May 29;41(7):1106–14. doi: 10.1093/humrep/deag075 (PMC13334914; doi:10.1093/humrep/deag075)
Supplement: deag075_Supplementary_Table_S3 [file deag075_supplementary_table_s3.pdf]

Human Reproduction, 2026, 1–1
https://doi.org/10.1093/humrep/deag075
Supplementary Data

human reproduction

OXFORD

Supplementary Table S3. Outcome model for probability of a live birth given a cleavage or blastocyst stage embryo transfer.

Variable*	Adjusted odds ratio (95% confidence interval)
**Intercept**	0.18 (0.15–0.21)
**ART clinic**	Random intercept term; $\chi_{53.0} = 28.8\ (P < 0.01)$;
**Female age**	Spline term; $\chi_{8.2} = 330.8\ (P < 0.01)$; see Supplementary Fig. S3A
**Tubal disease**	
No	0.0 (reference)
Yes	0.83 (0.65–1.05)
**Number of oocytes retrieved**	
1–2	0.0 (reference)
3–4	0.99 (0.85–1.15)
5 or more	0.82 (0.71–0.96)
**Use of testicular sperm**	
No	0.0 (reference)
Yes	0.87 (0.62–1.22)
**Fertilization method**	
IVF	0.0 (reference)
ICSI	0.91 (0.80–1.03)
**Stage of embryo development**	
Cleavage	0.74 (0.64–0.85)
Blastocyst	0.0 (reference)

For spline terms, we report the test statistic and *P*-value for the global test that the estimated effect is not everywhere zero. Comparison of cleavage and blastocyst stage transfer in patients with a single fertilized oocyte, data from Australia and New Zealand, 2009–2022.

* The coefficients for following variables were indistinguishable from zero as a result of ridge (L2) penalization: male age, endometriosis, male infertility, unexplained infertility, other female causes of infertility.
