# Supplementary material for: Cleavage stage versus blastocyst stage transfers in patients with a single zygote: an emulated target trial
Source: Hum Reprod. 2026 May 29;41(7):1106–14. doi: 10.1093/humrep/deag075 (PMC13334914; doi:10.1093/humrep/deag075)
Supplement: deag075_Supplementary_Table_S4 [file deag075_supplementary_table_s4.pdf]

Human Reproduction, 2026, 1–1
https://doi.org/10.1093/humrep/deag075
Supplementary Data

human reproduction OXFORD

Supplementary Table S4. Outcome model for probability of a clinical pregnancy given a cleavage or blastocyst stage embryo transfer.

**Variable***	**Adjusted odds ratio (95% confidence interval)**
**Intercept**	0.31 (0.27–0.36)
**ART clinic**	Random intercept term; $\chi_{53.0} = 15.2$ ($P < 0.01$);
**Female age**	Spline term; $\chi_{8.7} = 240.7$ ($P < 0.01$); see Supplementary Fig. S3A
**Male age**	Spline term; $\chi_{7.3} = 11.1$ ($P = 0.14$); see Supplementary Fig. S3B
**Tubal disease**	
No	0.0 (reference)
Yes	0.86 (0.70–1.06)
**Unexplained infertility**	
No	0.0 (reference)
Yes	0.89 (0.77–1.02)
**Number of oocytes retrieved**	
1–2	0.0 (reference)
3–4	0.95 (0.83–1.09)
5 or more	0.83 (0.73–0.95)
**Source of sperm**	
Ejaculate	0.0 (reference)
Testicular	0.91 (0.67–1.23)
**Stage of embryo development**	
Cleavage	0.74 (0.66–0.84)
Blastocyst	0.0 (reference)

For spline terms, we report the test statistic and *P*-value for the global test that the estimated effect is not everywhere zero. Comparison of cleavage and blastocyst stage transfer in patients with a single fertilized oocyte, data from Australia and New Zealand, 2009–2022.

* The coefficients for following variables were indistinguishable from zero as a result of ridge (L2) penalization: endometriosis, unexplained infertility, other female causes of infertility, male infertility.

© The Author(s) 2026. Published by Oxford University Press on behalf of European Society of Human Reproduction and Embryology.
This is an Open Access article distributed under the terms of the Creative Commons Attribution License (https://creativecommons.org/licenses/by/4.0/), which permits unrestricted reuse, distribution, and reproduction in any medium, provided the original work is properly cited.
Human Reproduction, 2026, 00, 1–18
https://doi.org/10.1093/humrep/deag075
Original Article
